# Acute systemic and energy metabolism responses to velocity‐based resistance training following an oral glucose load in individuals with excess body weight

**DOI:** 10.1113/EP093162

**Published:** 2025-12-11

**Authors:** Hugo Alejandro Carrillo‐Arango, Miguel Alejandro Atencio‐Osorio, Leidy Tatiana Ordoñez‐Mora, Baisuli Benitez‐Gómez, Robinson Ramírez‐Vélez, Mikel Izquierdo

**Affiliations:** ^1^ Grupo de Investigación en Deporte de Rendimiento (GRINDER), Programa de Educación Física y Deporte Universidad del Valle Cali Colombia; ^2^ Department of Health, Physiotherapy Program, Health and Movement Research Group Universidad Santiago de Cali Cali Colombia; ^3^ Navarrabiomed, Hospital Universitario de Navarra (HUN), Universidad Pública de Navarra (UPNA) Instituto de Investigación Sanitaria de Navarra (IdiSNA) Pamplona Spain; ^4^ CIBER of Frailty and Healthy Aging (CIBERFES) Instituto de Salud Carlos III Madrid Spain; ^5^ Facultad de Ciencias de la Educación Unidad Central del Valle del Cauca (UCEVA) Túlua Colombia

**Keywords:** dose–response, insulin sensitivity, metabolic response, physical activity, resistance training

## Abstract

We investigated the acute metabolic effects of two velocity‐based resistance training (RT) protocols, differing in intra‐set velocity loss (VL) thresholds, on postprandial substrate oxidation and glycaemic responses following a 75 g oral glucose tolerance test in individuals with excess body weight. A single‐group, randomized, cross‐over design was used, in which each participant completed three experimental conditions in random order: (1) control (rest); (2) RT with 20% velocity loss (VL20); and (3) RT with 40% velocity loss (VL40). Twenty‐four participants (50% female; median body mass index 30.2 kg m^−2^, interquartile range 27.9–34.1 kg m^−2^) were included in the final analysis. Each RT session consisted of bilateral leg‐press exercises at 55%–65% of one‐repetition maximum performed in four sets with 3 min rest intervals, while monitoring repetition velocity. Baseline measurements were performed in the fasted state (1012 h) with participants in the supine position for 30 min, after the oral glucose load at 60 min, and during the experimental conditions at 120, 180, and 240 min. Primary outcomes were respiratory quotient, oxygen uptake, carbon dioxide output, resting energy expenditure and substrate oxidation rates. Secondary outcomes included blood glucose, lactate, heart rate, power output and repetition volume. VL40 elicited greater cardiovascular and metabolic stress, evidenced by elevated heart rate and lactate levels (*p* < 0.0001). Both RT protocols decreased postprandial respiratory quotient compared with control conditions, with VL40 producing a larger shift towards fat oxidation (time × conditions interaction *p* < 0.0001). The glucose area under the curve was significantly lower in VL40 than in VL20 or control conditions (*p *< 0.0001). These findings suggest that velocity‐based RT acutely improves postprandial metabolism, with higher VL thresholds conferring superior fat oxidation and glycaemic regulation.

## Abstract (highlights)

- **What is the central question of this study?**
  Does the magnitude of intra‐set velocity loss during resistance training modulate acute postprandial energy metabolism and glycaemic control after an oral glucose load in individuals with excess body weight?
- **What is the main finding and its importance?**
  Resistance training performed to greater velocity loss thresholds enhanced fat oxidation and reduced postprandial glycaemia compared with control or lower velocity loss. These findings suggest that exercise intensity, operationalized through velocity loss, might optimize the acute metabolic benefits of resistance training in populations with excess adiposity.

## 1 INTRODUCTION

Obesity‐related non‐communicable diseases are driven by metabolic factors, including insulin resistance, visceral adiposity, immune dysregulation and impaired glucose homeostasis (Bae et al., 2024). Excess adiposity is an independent risk factor for type 2 diabetes mellitus, cardiovascular disease (Ford, 2005) and several types of cancers (Zhang et al., 2023), contributing to increased morbidity (Arpón et al., 2019) and mortality (Blüher, 2019).

Western dietary patterns, particularly those high in refined carbohydrates, trigger repeated postprandial excursions in glucose and insulin, along with prolonged lipaemia. These responses exacerbate oxidative stress and activate inflammatory pathways (Huang et al., 2022). In individuals with obesity, postprandial metabolic disturbances are intensified by chronic low‐grade inflammation and delayed metabolic recovery. Although postprandial hyperglycaemia promotes pro‐inflammatory responses, whether directly attenuating postprandial hyperglycaemia reduces inflammation remains unclear (Mazidi et al., 2021). Alternatively, whether cumulative prandial inflammatory episodes underlie disease progression (Manning et al., 2008), evidence suggests that postprandial hyperglycaemia and insulin excursions are stronger predictors of cardiometabolic risk than fasting hyperglycaemia, particularly in individuals with insulin resistance or excess adiposity (Bellini et al., 2021). Notably, elevated 2 h postprandial glucose levels have been associated with a 50% greater risk of cardiovascular events and an 89% increase in all‐cause mortality (Cavalot et al., 2011). Given that most of the day is spent in the postprandial state, strategies targeting postprandial hyperglycaemia are crucial for the prevention of cardiometabolic diseases.

Physical activity (any bodily movement produced by skeletal muscles resulting in energy expenditure) improves postprandial glucose and insulin regulation in healthy and insulin‐resistant individuals (Bird & Hawley, 2017). Exercise training (a structured and planned form of physical activity performed to improve fitness) promotes insulin‐independent glucose uptake via contraction‐mediated pathways (Conn et al., 2014) and enhances insulin sensitivity, both acutely and chronically. Resistance training (RT), in particular, improves glycaemic control for ≤72 h postexercise and induces sustained metabolic adaptations with repeated bouts (Roberts et al., 2013).

Traditionally, RT intensity is prescribed based on the percentage of the one‐repetition maximum (1RM), with the volume manipulated using the total number of sets and repetitions (Izquierdo et al., 2006; Sánchez‐Medina & González‐Badillo, 2011). Recently, intra‐set velocity loss (VL) has emerged as a reliable indicator of neuromuscular fatigue and volume load (Izquierdo et al., 2006; Jukic et al., 2023). Intra‐set VL is correlated with mechanical and metabolic fatigue markers, in addition to proximity to muscular failure (Gorostiaga et al., 2012). For example, terminating a set at 20% reflects moderate fatigue (∼50% of maximal repetitions completed), whereas 40%–50% of the VL approaches volitional failure (Izquierdo et al., 2006; Pareja‐Blanco et al., 2017). Thus, the VL offers a precise tool for regulating RT prescriptions beyond traditional loading parameters (Pareja‐Blanco et al., 2017; Sánchez‐Medina & González‐Badillo, 2011).

Excess adiposity impairs postprandial substrate oxidation and insulin sensitivity, contributing to metabolic inflexibility and cardiometabolic risk (Bird & Hawley, 2017). RT has been shown to enhance energy expenditure and glucose regulation, yet the acute systemic metabolic responses can vary according to the degree of neuromuscular fatigue incurred during exercise (Pareja‐Blanco et al., 2017; Sánchez‐Medina & González‐Badillo, 2011). Velocity‐based RT, which quantifies effort through intra‐set VL, provides a mechanistic means to control exercise intensity and metabolic stress. Despite robust evidence for exercise‐mediated improvements in glycaemic regulation, the optimal timing and intensity of RT in the postprandial period remain unclear. We hypothesized that higher intra‐set VL would elicit greater acute metabolic and cardiovascular responses, characterized by increased oxygen consumption, enhanced fat oxidation and improved postprandial glucose regulation compared with lower VL and rest conditions. Accordingly, the aims of this study were as follows: (1) to compare the acute effects of two RT protocols differing in VL thresholds (20% vs. 40%) on postprandial substrate oxidation and energy metabolism; and (2) to examine the impact of these RT protocols on postprandial glycaemic and lactate responses, following a 75 g oral glucose tolerance test in individuals with excess body weight.

## 2 MATERIALS AND METHODS

### 2.1 Ethical approval

This study was conducted in accordance with the *Declaration of Helsinki* (2013 revision) and received approval from the Ethics Committee for Research with Human Subjects of the University of Valle Ethics Committee (ID numbers 174‐020 and 018‐020). All participants were fully informed about the study procedures, potential risks and benefits before participation and provided written informed consent prior to enrolment.

### 2.2 Participants and setting

A total of 24 participants were included in the final analysis (50% female), with a median body mass index of 30.2 kg m^−2^ (interquartile range = 27.9–34.1 kg m^−2^), recruited through advertisements and by contacting volunteers listed at the University of Valle. Eligible participants had no known history of cardiovascular or renal disease, diabetes or any other condition that could pose a risk during participation in this protocol. The exclusion criteria were any known disease, orthopaedic or neurological limitations to exercise, surgery during the intervention period, drug or alcohol abuse (determined using the FANTASTIC survey) (Ramírez‐Vélez & Agredo, 2012), medication use, and pregnancy (in women). All participants engaged in activities of daily living and recreation but did not routinely participate in physical exercises. The prevalence of self‐reported tobacco use was 8.3%; none of the participants met the recommendation of 150 min per week of physical activity or spent >8 h per day in sedentary behaviour. This trial was conducted from June 2024 to July 2025 at the University of Valle, Cali, Colombia.

### 2.3 Study design

**FIGURE 1:**
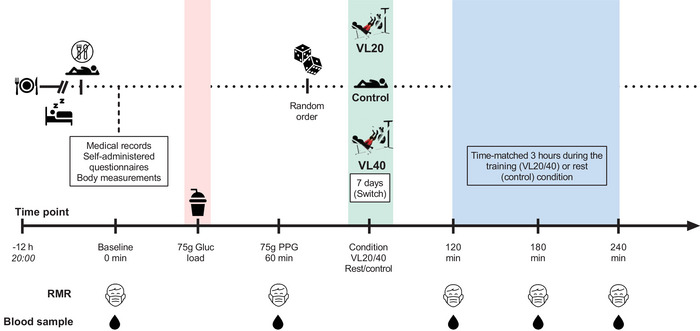
Overview of experimental protocol. Participants arrived at the laboratory following a 10‐12 h overnight fast and were instructed to avoid unusual physical activity, alcohol and caffeine for 48 h before testing. Upon arrival (time 0 min), medical history, self‐administered questionnaires and anthropometric measurements were obtained. Baseline resting metabolic rate (RMR) and a capillary blood sample were collected before the ingestion of a 75 g oral glucose load. At 60 min post‐glucose ingestion, participants began one of the three experimental conditions. The resistance training sessions (VL20 and VL40) were performed on a leg‐press machine in standardized conditions, with exercise intensity and volume controlled by monitoring velocity loss (VL) during the concentric phase of each repetition using a linear position transducer. In the VL20 conditions, each set was terminated when mean concentric velocity decreased by 20% from the fastest repetition, whereas in VL40 conditions the set continued until a 40% velocity loss was reached, representing a greater degree of fatigue and total workload. The control conditions involved time‐matched rest. Subsequent RMR and blood samples were obtained at 120, 180 and 240 min following the start of each of the conditions. All experimental procedures were performed at the same time of day to account for diurnal variation in metabolic and hormonal responses. This cross‐over design enabled each participant to serve as their own control, thereby reducing interindividual variability and improving the sensitivity for detecting differences in metabolic and physiological responses to varying levels of resistance exercise‐induced fatigue. Face‐mask icons represent indirect calorimetry (metabolic cart) assessments. The soda cup icon represents a 75 g oral glucose dose. Postprandial plasma glucose (PPG). Drop icons represent capillary blood sample assessment.

This study used a single‐group, randomized, cross‐over experimental design, in which each participant completed three experimental conditions in random order: (1) control (rest); (2) VL20; and (3) VL40 following a 75 g oral glucose tolerance test. The trials were separated by ≥7 days to minimize potential testing bias and included assessments of anthropometric and body composition measurements, individual load–velocity relationships and 1RM load. The time line for each trial is shown on the *x*‐axis in Figure 1. Each trial began at 07.00 h after an overnight fast (10‐12 h) to minimize diurnal biological variation. Participants were instructed to avoid caffeine, alcohol and unusual physical activity for 2 days before performing the test. The duration of the entire session was ∼6 h.

Baseline measurements were performed in the fasted state (10–12 h) in the supine position for 30 min before oral glucose load and exercise. Participants then ingested the standard solution used for the oral glucose tolerance test (75 g of glucose in 250 mL, Dextrosol HYCEL; Grupo JAFS, Culiacán, Mexico) and returned to the supine position immediately for 1 h. Complementary measurements were obtained after the oral glucose load at 60 min, and during the experimental conditions at 120, 180, and 240 min. The thermal feedback device was maintained at room temperature (24°C ± 1°C).

### 2.4 Preliminary testing: Visit 1

Volunteers reported to a laboratory at the University of Valle to complete the Physical Activity Readiness Questionnaire (PAR‐Q+) (Warburton et al., 2014), complete screening and medical information forms. All physiological measurements were conducted following standardized procedures. Resting blood pressure and heart rate were measured in a seated position after ≥10 min of rest using an automated digital sphygmomanometer (Omron HEM705 CP; Omron Healthcare UK Ltd, Milton Keynes, UK). Two readings were taken 1 min apart, and the average value was recorded. Body mass (in kilograms) and height (in metres) were measured using a balance scale (Seca 284; SECA, Hamburg, Germany), and the body mass index was calculated as weight/height^2^ (in kilograms per metre squared). Body composition was assessed using tetrapolar bioimpedance analysis (BC‐418 MA; Tanita Corp., Tokyo, Japan). Capillary blood samples were collected to measure the concentrations of lactate (Lactate Pro 2 Meter LP2; Arkray Inc., Kyoto, Japan), glucose (Accutrend Plus portable; Roche Diagnostic Australia Pty Ltd, Castle Hill, NSW, Australia), and fasting ketone bodies (β‐hydroxybutyrate; Freestyle Optium Neo^®^; Abbott Laboratories Inc., Abbott Park, IL, USA) using commercially available devices. The resting metabolic rate was measured using a Q‐NRG+ calorimeter (COSMED, Albano Laziale, Italy), a validated system for assessing the volume of carbon dioxide production (${\dot{V}}_{CO_{2}}$), oxygen consumption (${\dot{V}}_{O_{2}}$), resting energy expenditure (REE), respiratory quotient (RQ) and the rate of utilization of fat and carbohydrates. During the test, a ventilated plastic canopy connected to the instrument was placed over the participant to allow normal breathing. The ${\dot{V}}_{CO_{2}}$ and ${\dot{V}}_{O_{2}}$ levels in the inhaled and exhaled air were measured every 5 s using an indirect calorimeter for 30 min. The flow sensor and gas analysers were calibrated using gases of known concentrations (16% O_2_ and 5% CO_2_) and volumes (3 L syringe) before each test. The same gas analyser was used for all the tests. The first 5 min of data were discarded, and the average ${\dot{V}}_{CO_{2}}$ (in millilitres per minute) and ${\dot{V}}_{O_{2}}$ (in millilitres per minute) were calculated over a 10 min stable period. Fat and carbohydrate utilization were estimated using the equation described by Frayn (1983). For the analysis of substrate metabolism, urinary nitrogen excretion was zero for all calculations.

- Fat oxidation rate (g min^−1^) = 1.67 × ${\dot{V}}_{O_{2}}$ (L min^−1^) − 1.67 × ${\dot{V}}_{CO_{2}}$ (L min^−1^)
- Carbohydrate oxidation rate (g min^−1^) = 4.55 × ${\dot{V}}_{CO_{2}}$ (L min^−1^) − 3.21 ${\dot{V}}_{O_{2}}$ (L min^−1^)
- Lipid oxidation rate/body mass (mg kg^−1^ min^−1^) = 1.6946 × ${\dot{V}}_{O_{2}}$ (mL kg^−1^ min^−1^) − 1.7012 × ${\dot{V}}_{CO_{2}}$ (mL kg^−1^ min^−1^)
- Lipid energy output (kcal kg^−1^ min^−1^) = Lipid oxidation rate (mg kg^−1^ min^−1^) × 9
- Carbohydrate oxidation rate/body mass (mg kg^−1^ min^−1^) = 4.5850 × ${\dot{V}}_{CO_{2}}$ (mL kg^−1^ min^−1^) − 3.2255 × ${\dot{V}}_{O_{2}}$ (mL kg^−1^ min^−1^)
- Total energy output (kcal kg^−1^ min^−1^) = Lipid oxidation rate (mg kg^−1^ min^−1^) × 9 + Carbohydrate oxidation rate (mg kg^−1^ min^−1^) × 4
- REE (kcal/day) = 3.9 × ${\dot{V}}_{CO_{2}}$ (mL min^−1^) + 1.1 × ${\dot{V}}_{O_{2}}$ (mL min^−1^) × 1440
- Fat substrate rate (%) = [(1 − RQ)/0.29] × 100
- Carbohydrate substrate rate (%) = [(RQ − 0.71)/0.29] × 100

If the RQ was >1, fat oxidation was considered zero, and the data were calculated accordingly (Farinatti et al., 2016; Vianna et al., 2014).

### 2.5 Progressive loading testing: Visit 2

Before the evaluations, all participants performed a general 10 min warm‐up of pedalling on a stationary bicycle (50–70 r.p.m.; resistance levels 1–5 on the 10‐point Borg scale). Subsequently, they performed a progressive loading test of 1RM in the bilateral leg‐press exercise (EVOST Fitness Line, dimensions 220 cm × 163 cm × 130 cm; MoviFIT, Valle, Colombia). The machine features a 45° tilt for the lower body, a 30° tilt for the trunk, a minimum load of 25 kg, and hands placed on the side handles. After receiving the instructions, the participants performed a purely concentric action and slowly returned to their initial position before repeating the exercise. Both the concentric and eccentric phases were controlled by an experienced researcher who placed their hands on the handle of the platform. To be considered a valid repetition, knee flexion had to exceed ∼90° during the eccentric phase, and the movement was completed with the knees extended in the concentric phase. This position was recorded for each participant and marked such that the evaluator provided an audible signal upon reaching the final position. All participants received verbal encouragement to perform the concentric phase as quickly and forcefully as possible against all weights. The range of motion and velocity values for all repetitions were recorded at 1000 Hz using a linear velocity transducer (T‐Force Dynamic Measurement System, Ergotech Consulting, Murcia, Spain), the reliability of which has been reported previously (Sánchez‐Medina & González‐Badillo, 2011). To estimate the 1RM load, sex‐specific equations were used for the leg‐press exercise (Marcos‐Pardo et al., 2019).

During each repetition, the peak velocity (maximum instantaneous velocity value reached during the concentric phase) and mean velocity (average velocity from the start of the concentric phase until the weight stack plate reached the maximum height) were displayed in real time using custom software (T‐Force Dynamic Measurement System). The fastest peak and mean velocity values for each weight were analysed, including the baseline load displaced at the maximum intended velocity. The warm‐up consisted of two sets of five repetitions with weights ranging from 30 to 40 kg. The initial load during the test was set to 25 kg for all participants. Similar to a previous study (Marcos‐Pardo et al., 2019), the load was increased gradually, initially in 20 kg increments, until a mean propulsive velocity of ∼0.30 m s^−1^ was reached. Strong verbal encouragement was provided during the tests to motivate the participants to exert maximal effort. Only the repetition with the highest maximum velocity for each load was considered for further analysis. A 3 min rest was provided between sets.

### 2.6 Acute RT session

Each session began with a 10 min warm‐up, pedalling on a stationary bicycle (50–70 r.p.m.; resistance levels 1–5 on the 10‐point Borg scale). The participants performed a specific warm‐up consisting of five repetitions at 60% of their training load. During the first set, the set was stopped to adjust the load if the velocity reached in the first three repetitions did not match the programmed velocity (±0.03 m s^−1^). Once adjusted, the load was maintained for all the sets. For each session, the total repetitions per set, fastest mean velocity, average mean velocity and average VL were analysed separately. The participants did not engage in any purposeful exercise on the control, non‐exercise day. Water was provided ad libitum during passive rest intervals, and the participants were hydrated after the exercise period. All participants tolerated the exercise regimen well; however, one participant experienced nausea during the VL40 session and completed only three sets of exercises. The mean room temperature and relative air humidity (*p* = 0.50) were similar during the exercise sessions in both trials.

### 2.7 Statistical analyses

All statistical analyses were performed using SPSS v.26.0 (IBM Corp., Armonk, NY, USA), and figures were generated with GraphPad Prism v.8.0 (GraphPad Software Inc., San Diego, CA, USA). Statistical significance was set at *p* < 0.05. Participant characteristics are presented as the mean ± SD, median (25th–75th percentile) or number (percentage), unless otherwise indicated. The normality of distributions was assessed using the Ryan–Joiner test. A two‐way repeated‐measures ANOVA was used to examine the effects of time (fasting, after the oral glucose load at 60 min, and during the experimental conditions at 120, 180, and 240 min) and (control, VL20 and VL40). Time was treated as a within‐subject factor and condition as a between‐subject factor. The time × condition interaction was evaluated to determine whether changes over time differed among trials. When significant main or interaction effects were detected, Fisher's least significant difference (LSD) *post hoc* tests were applied to control type I error. Effect sizes were estimated using partial eta‐squared (ηp^2^) values, interpreted as small (0.04), medium (0.25) and large (0.64). Sphericity was tested using Mauchly's test, and when violated, Greenhouse–Geisser corrections were applied. The area under the curve (AUC) for metabolic variables was calculated using the trapezoidal rule. Differences in AUC between experimental conditions were examined using one‐way ANOVA with Bonferroni *post hoc* correction. Sensitivity analyses were conducted using the same repeated‐measures mixed models, stratified by sex (men and women analysed separately).

## 3 RESULTS

### 3.1 Participant characteristics

The baseline characteristics are presented in Table 1. Despite being overweight or obese and physically inactive, the participants demonstrated relatively preserved metabolic profiles. Except for high‐density lipoprotein cholesterol, low‐density lipoprotein cholesterol and glucose concentrations, all other blood parameters remained within the reference ranges for age‐matched healthy adults.

**TABLE 1:** Characteristics of participants at rest and fasting.

Characteristics	Sample (*n *= 24)
Anthropometric and body composition parameters	
Age, years	25.5 (24.0–30.0)
Body mass, kg	93.6 (73.5–99.4)
Height, m	1.66 (1.59–1.77)
Body mass index, kg m^−2^	30.2 (27.9–34.1)
Waist circumference, cm	91.7 (81.3–98.8)
Body fat, %	31.1 (24.6–42.1)
Fat‐free mass, kg	58.3 (46.2–71.2)
Clinical and metabolic parameters	
Systolic blood pressure, mmHg	125.5 (119.8–130.0)
Diastolic blood pressure, mmHg	77.5 (71.3–85.3)
Mean blood pressure, mmHg	94.0 (89.1–99.3)
Heart rate, beats min^−1^	65.0 (59.0–72.3)
Triglycerides, mg dL^−1^	108.2 (74.0–134.5)
Cholesterol, mg dL^−1^	165.0 (146.3–185.0)
HDL, mg dL^−1^	31.5 (24.5–37.5)
LDL, mg dL^−1^	111.0 (101.0–113.6)
Glucose, mg dL^−1^	109.0 (103.3–113.5)
Lactate, mmol L^−1^	1.7 (1.4–2.0)
Ketone bodies (β‐hydroxybutyrate), mg dL^−1^	0.42 (0.40–0.48)
Resting energy expenditure, kcal	1775.0 (1493.5–1968.0)
Total energy output, kcal kg^−1^ min^−1^	13.7 (12.6–14.9)
Respiratory quotient, a.u.	0.78 (0.73–0.84)
Oxygen consumption, mL kg^−1^ min^−1^	2.9 (2.8–3.1)
Carbon dioxide production, mL kg^−1^ min^−1^	2.3 (2.0–2.5)
Fat substrate rate, %	75.9 (54.3–92.3)
Carbohydrate substrate rate, %	24.1 (7.8–45.7)
Fat utilization rate, mg kg^−1^ min^−1^	1.1 (0.7–1.2)
Carbohydrate rate, mg kg^−1^ min^−1^	0.9 (0.4–1.8)
Fat ox/fat‐free mass, mg (kg FFM) ^−1^ min^−1^	1.6 (1.3–1.9)
CH ox/fat‐free mass, mg (kg FFM)^−1^ min^−1^	1.5 (0.6–2.9)
Lipid energy output, kcal kg^−1^ min^−1^	9.6 (6.6–11.2)
Lifestyle parameters, *n* (%)	
Tobacco (≥10 cigarettes per week)	2 (8.3)
Alcohol (1–2 times per week)	4 (17.1)
Self‐reported morbidity, *n* (%)	
Chronic low back pain	2 (8.3)
Respiratory allergy/asthma/others	3 (12.5)
Headache	2 (8.3)
Self‐reported ethnicity, *n* (%)	
Afro descendent	7 (29.2)
Mestizo	9 (37.5)
Others	8 (33.3)

*Note*: Continuous variables are expressed as the median (25th−75th percentile value) and categorical variables as the frequency (percentage). Self‐administered questionnaires were distributed to 24 participants to obtain information about demographic characteristics, lifestyle and morbid disorders confirmed by clinical diagnosis by a physician. Lifestyle parameters were measured using a self‐report questionnaire. Habits, such as smoking cigarettes (not smoking and those who currently smoke ≥10 cigarettes per week) and alcohol consumption (drinking on <1 day per week or no alcohol intake), were evaluated. Abbreviations: CH, carbohydrate; FFM, fat‐free mass; HDL, high‐density lipoprotein; LDL, low‐density lipoprotein; ox, oxidation.

### 3.2 Resistance training measurements

**FIGURE 2:**
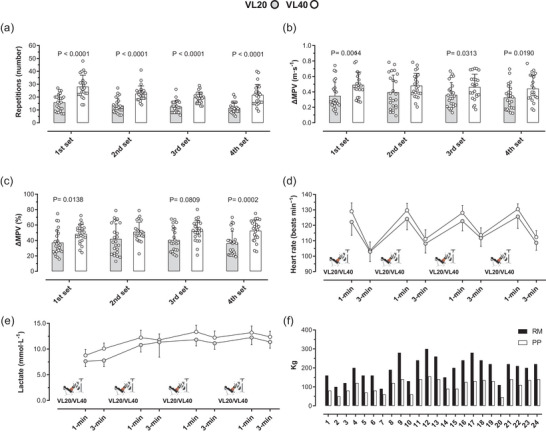
Training performance and acute physiological responses during the velocity‐based resistance training protocols. (a) Number of repetitions performed per set. (b) Absolute mean propulsive velocity (ΔMPV; in metres per second) per set. (c) Relative change in mean propulsive velocity (ΔMPV; as a percentage). (d) Heart rate responses (in beats per minute) recorded 1 and 3 min after each set. (e) Blood lactate concentration (in millimoles per litre) measured 1 and 3 min after each set. (f) Individual values of repetition maximum (RM) and peak power (PP) during leg‐press exercise. Data are presented as the mean (SD).

All the participants completed the RT protocols. One participant discontinued the VL40 session owing to nausea after completing three of four sets, but their data were retained in the analysis. The average training volume across the four sets was 13 ± 2 repetitions per set (total repetitions = 1248) for VL20 and 22 ± 4 repetitions per set (total repetitions = 2112) for VL40. Assuming a mean load of 60% 1RM (∼120 kg), the total estimated lifted volume was 149 760 ± 26 956 kg for VL20 and 253 440 ± 45 619 kg for VL40. These values are consistent with the 15%–20% interindividual variability typically observed in velocity‐based resistance training protocols (Pareja‐Blanco et al., 2017; Sánchez‐Medina & González‐Badillo, 2011; Figure 2a). Performance metrics, including power output, work completed, heart rate, blood glucose and lactate, are shown in Figure 2b−f.

The RT protocol consisted of four sets at the maximal intended velocity, with ∼3 min of inter‐set recovery. The average decline in the mean propulsive velocity (Δvelocity) across sets was as follows: VL20, 0.35, 0.35, 0.36 and 0.33 m s^−1^ (relative changes, 37%–41%); and VL40, 0.49, 0.48, 0.46 and 0.44 m s^−1^ (relative changes, 50%–52%) (Figure 2b, c). Heart rate, monitored continuously, ranged between 103 and 130 beats min^−1^ during RT and followed the expected recovery kinetics (*p* < 0.0001; Figure 2d). Capillary blood lactate, sampled 1 and 3 min post‐set, increased 6‐ to 12‐fold from baseline (mean baseline = 1.7 ± 0.3 mmol L^−1^), reaching 7.6–13.3 mmol L^−1^ across sessions (*p* < 0.0001; Figure 2d). Baseline strength testing revealed 1RM values (defined as mean propulsive velocity < 0.30 m s^−1^) ranging from 152 to 240 kg and peak power output between 80 and 138 kg (Figure 2f).

### 3.3 Respiratory quotient, resting energy expenditure and metabolic gases

**FIGURE 3:**
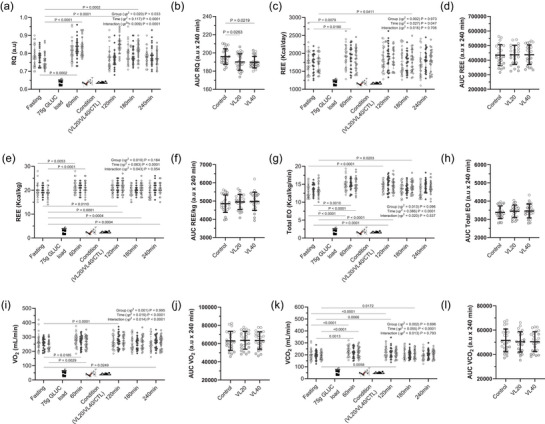
Energy metabolism responses following oral glucose ingestion in different experimental conditions (control, VL20 and VL40). Time course of changes in: (a, b) respiratory quotient (RQ); (c, d) resting energy expenditure (REE) kcal/day; (e, f) resting energy expenditure (REE) kcal/min; (g, h) total energy oxidation (TEO); (i, j) oxygen uptake (V_O2_); and (k,l) carbon dioxide output (VCO_2_) across the three experimental conditions: control (rest), low‐velocity loss RT (VL20) and high‐velocity loss RT (VL40); (b,d,f,h,j,l) area under the curve (AUC) for energy metabolism responses. Control (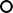 *n *= 24); VL20 (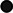 Velocity loss 20%, *n *= 24); VL40 (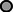 Velocity loss 40%, *n *= 24) conditions. All values are reported as the mean (SD). For AUC, each data point represents one participant (*n *= 24, ♀ grey circles, *n *= 12; ♂ white circles, *n *= 12). Bars denote the mean (SD).

Figure 3 illustrates the postprandial responses to a 75 g oral glucose load across the conditions. The respiratory quotient increased significantly in both the VL20 (*p* = 0.002) and VL40 conditions (*p* < 0.0001). Main effects were detected for time (*p* < 0.0001, ηp^2^ = 0.117) and conditions (*p* = 0.033, ηp^2^ = 0.020), with a significant time × conditions interaction (*p *< 0.0001, ηp^2^ = 0.009). The AUC for RQ was lower in both RT conditions than in the control conditions (VL20 *p* = 0.0263 and VL *p* = 0.029; Figure 3a, b).

Resting energy expenditure increased over time (*p* = 0.047, ηp^2^ = 0.027); however, it showed no significant group or interaction effects (Figure 3c). These results persisted after adjusting for fat‐free mass (REE/FFM), with no conditions differences in the AUC (Figure 3d, f). The total energy expenditure increased significantly over time (*p *< 0.0001, ηp^2^ = 0.086). However, there was no difference between the conditions (Figure 3g). Both oxygen uptake and carbon dioxide output increased postprandially (*p *< 0.0001 for time), with time × conditions interactions indicating higher early oxygen uptake responses in the VL40 conditions (Figure 3i, k). However, the AUC comparisons were not statistically different across the conditions (Figure 3h, j, l).

### 3.4 Fat and carbohydrate oxidation

**FIGURE 4:**
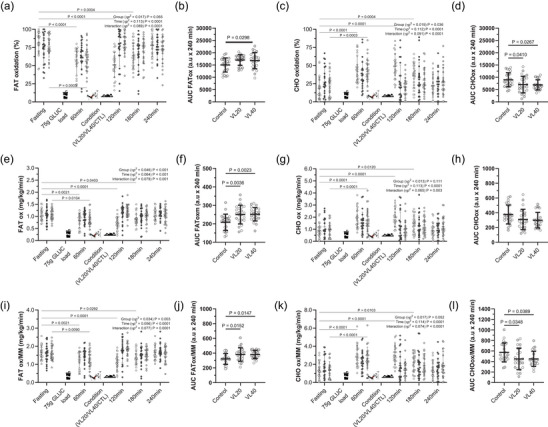
Substrate oxidation responses following oral glucose ingestion in different experimental conditions (control, VL20 and VL40). Time course of changes in: (a, c) percentage contribution of fat and carbohydrate oxidation over time; (e, g) absolute fat and carbohydrate oxidation rates (in milligrams per kilogram per minute); (i, k) fat and carbohydrate oxidation rates normalized to muscle mass (in milligrams per kilogram per minute); (b,d,f,h,j,l) area under the curve (AUC) for total and muscle mass‐adjusted fat and carbohydrate oxidation. Control (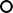 *n *= 24); VL20 (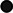 velocity loss 20%, *n *= 24); VL40 (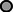 velocity loss 40%, *n *= 24) conditions. All values are reported as the mean (SD). For AUC, each data point represents one participant (*n* = 24, ♀ grey circles, *n *= 12; ♂ white circles, *n *= 12). Bars denote the mean (SD).

Postprandial substrate oxidation is shown in Figure 4. Fat oxidation (as a percentage) increased over time across all conditions (*p *< 0.0001, ηp^2^ = 0.113), with a trend towards higher values in the VL40 conditions (Figure 4a). AUC analysis confirmed significantly greater fat oxidation in the VL40 conditions than in the control conditions (*p* = 0.029; Figure 4b). Carbohydrate oxidation (as a percentage) showed significant main effects of conditions (*p* = 0.036, ηp^2^ = 0.019), time (*p* < 0.0001, ηp^2^ = 0.112) and interaction (*p *< 0.0001, ηp^2^ = 0.091). VL40 exhibited a steeper decline in carbohydrate oxidation during the early postprandial phases (Figure 4c). AUCs were significantly greater in both the VL20 and VL40 conditions than in the control conditions (*p* = 0.026 and *p* = 0.041, respectively; Figure 4d). When expressed in milligrams per kilogram per minute and normalized to FFM, fat oxidation demonstrated a significant time × conditions interaction (*p* < 0.0001, ηp^2^ = 0.079), with VL40 maintaining the highest rate during recovery (Figure 4e). AUC analysis supported greater fat oxidation in both absolute and relative terms for VL40 (*p* = 0.0023 and *p* = 0.0147, respectively; Figure 4f,j). Likewise, carbohydrate oxidation (relative in milligrams per kilogram per minute) was lower in both the VL20 and VL40 conditions than in the control, with statistically significant differences in AUC (*p* = 0.034 and *p* = 0.039, respectively; Figure 4l).

### 3.5 Blood and cardiovascular variables

**FIGURE 5:**
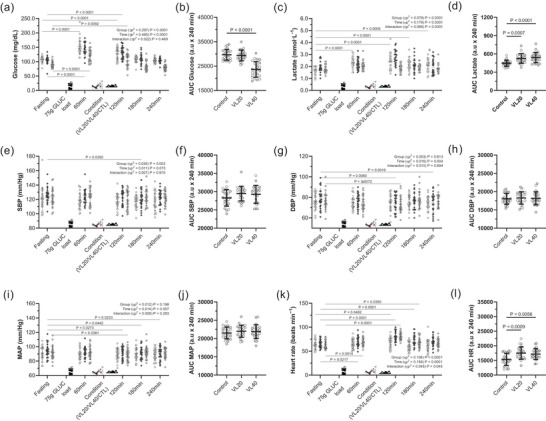
Cardiometabolic and haemodynamic responses following oral glucose ingestion in different experimental conditions (control, VL20 and VL40). Time course of changes in glucose (a), lactate (c), systolic (e), diastolic (g) and mean arterial pressure (i) and heart rate (k) following a 75 g oral glucose load in control, VL20 and VL40 conditions. Corresponding AUC comparisons (b, d, f, h, j, l) are presented next to each time course panel. Heart rate (HR; Polar H10 HR monitor, Polar Electro Oy, Kempele, Finland) and blood pressure (Omron Healthcare Co., Ltd, Kyoto, Japan) were recorded at the end of every stage (i.e. last 15 s of each stage). Control (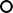 *n *= 24); VL20 (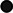 velocity loss 20%, *n *= 24); VL40 (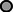 velocity loss 40%, *n *= 24) conditions. All values are reported as the mean (SD). For the AUC, each data point represents one participant (*n* = 24, ♀ grey circle, *n *= 12; ♂ white circle, *n *= 12). Bars denote the mean (SD).

Figure 5 shows the time course of plasma glucose responses following an oral glucose load. Glucose concentrations increased sharply across all conditions, peaking at 60 min post‐ingestion (*p* < 0.0001) and declining thereafter (Figure 5a). Both RT protocols attenuated the glucose response relative to the control conditions (main effect of conditions: *p *< 0.0001, ηp^2^ = 0.297), with significantly lower AUCs observed in control conditions compared with the VL40 conditions (*p* < 0.0001). Postprandial lactate concentrations also increased significantly, particularly in the VL40 conditions (*p *< 0.0001), indicating greater glycolytic flux during the recovery period (Figure 5c). The lactate AUC was significantly elevated in the VL40 conditions compared with the control conditions (*p* < 0.0001; Figure 5d). Systolic blood pressure (SBP) decreased modestly over time (*p* = 0.073, ηp^2^ = 0.011). However, no significant conditions or AUC effects were detected (Figure 5f). Diastolic blood pressure (DBP) and mean arterial pressure (MAP) remained stable, with only minor time effects (*p* = 0.004 and *p* = 0.007, respectively) and no significant differences between conditions (Figure 5g–j).

Heart rate showed significant conditions (*p* < 0.0001, ηp^2^ = 0.106) and time effects (*p *< 0.0001, ηp^2^ = 0.159). Both VL40 and VL20 induced greater elevations during the early recovery phase compared with the control conditions (Figure 5k). AUC analysis confirmed significantly higher heart rate in the VL40 than in the control (*p* = 0.0056) and VL20 conditions (*p* = 0.0009; Figure 5l).

### 3.6 Sensitivity analyses: Effects of RT protocols by sex

Sex‐stratified results (Figures 3, 4, 5; AUC panels) revealed consistent intervention effects across both sexes. Men exhibited higher absolute values of resting energy expenditure, ${\dot{V}}_{O_{2}}$ and ${\dot{V}}_{CO_{2}}$ in both VL20 and VL40 conditions (all *p* < 0.0001). In contrast, women demonstrated significantly higher relative fat oxidation during the VL20 protocol (*p* = 0.0257). No other major sex × condition interactions were found.

## 4 DISCUSSION

This study is the first to examine the acute effects of contraction intensity, manipulated through VL thresholds, on systemic and energy postprandial metabolism following RT in individuals with excess body weight. Using a controlled bilateral 45° leg‐press protocol performed immediately before a standardized 75 g oral glucose tolerance test, we observed that a higher VL threshold (40%) elicited superior metabolic adaptations compared with both a lower threshold (20%) and a non‐exercise control. Specifically, the VL40 conditions reduced postprandial glycaemic excursions and increased fat oxidation, indicating improved metabolic flexibility and substrate utilization. These findings highlight the potential of manipulating VL as a practical training variable to optimize the acute metabolic benefits of resistance exercise in individuals with excess body weight.

Our findings support the utility of whole‐body calorimetry in capturing short‐term substrate utilization dynamics after moderate RT exercise in adults with excess body weight. Importantly, VL40 resulted in more favourable postprandial glycaemic control and substrate oxidation patterns than VL20, with pronounced reductions in glucose AUC and increases in relative fat oxidation. These results are clinically relevant, given that impaired postprandial glucose tolerance and blunted lipid oxidation are key features of insulin resistance and the development of cardiometabolic disease (Dimina & Mariotti, 2019).

As expected, ingestion of a 75 g glucose load led to incresases in plasma glucose, blood lactate, RQ, REE and total energy expenditure. These changes reflect a metabolic shift towards carbohydrate oxidation driven by elevated insulin concentrations during the postprandial state (Heden et al., 2015; Kiens, 2006). However, prior exercise, particularly with a greater VL, modulated these responses. VL40 caused prolonged increases in REE and ${\dot{V}}_{O_{2}}$ remained elevated up to 180 min postexercise, while VL20 effects were persisted up to 120 min, indicating dose‐dependent effects of RT on postprandial energy metabolism.

The strength of this study lies in its matched design across protocols: exercise variables (load, set structure, inter‐set rest, cadence and range of motion) were tightly standardized, with the VL thresholds used as the only manipulated variable. This enabled for the isolation of training volume (i.e. number of repetitions), which was approximately nine repetitions higher in the VL40 group than in the VL20 group. Despite similar gross energy expenditure during the sessions, the metabolic adaptations differed, highlighting that training volume per se, when paired with sufficient intensity, might enhance post‐exercise substrate oxidation.

Interestingly, although REE and total energy output increased in both RT conditions, the differences between VL20 and VL40 were more prominent in fat oxidation than in carbohydrate oxidation. This supports the idea that moderate‐ to high‐intensity RT enhances lipid metabolism during the recovery phase, even with postprandial hyperinsulinaemia, a known inhibitor of lipolysis (Ramírez‐Vélez et al., 2025).

The RQ responses confirmed that both VL protocols promoted mixed‐substrate oxidation, with energy derived from both fatty acids and glucose. Although the absolute and relative fat and carbohydrate oxidation rates were broadly similar between VL20 and VL40, the lower RQ and elevated fat oxidation following VL40 suggest a metabolic shift favouring lipid utilization during recovery. This might reflect increased skeletal muscle AMPK activation and mitochondrial oxidative capacity (Heden et al., 2015; Kiens, 2006; Ramírez‐Vélez et al., 2025).

Our findings align with those of previous studies, which demonstrate reduced RQ after exercise in individuals with type 2 diabetes mellitus (Nygaard et al., 2017). However, they differ from reports showing no effect of exercise timing on substrate oxidation (Ferguson et al., 2001; Terada et al., 2016). Discrepancies across studies are probably due to stem from heterogeneity in participant characteristics, such as age, metabolic status and medication use, which are not present in our cohort.

Interestingly, previous studies have reported that fast‐twitch, high‐velocity contractions can produce greater increases in REE and ${\dot{V}}_{O_{2}}$ (Mitchell et al., 2024; Terada et al., 2016), whereas postprandial exercise has been associated with reduced fat oxidation (Schneiter et al., 1995). These inconsistencies highlight the complexity of exercise timing and intensity as factors influcencing substrate metabolism. In our study, both RT protocols resulted in similar comparable substrate oxidation responses; however, VL40 showed a more sustained increase in energy expenditure, suggesting a potential advantage for metabolic recovery.

Sex‐specific analyses showed higher REE, ${\dot{V}}_{O_{2}}$ and ${\dot{V}}_{CO_{2}}$ values in men during both VL protocols, while they demonstrated greater relative fat oxidation during VL20. These patterns are consistent with the existing literature, which indicates that body composition, particularly FFM, has a strong influcence on energy metabolism (Cano et al., 2022; Pontzer et al., 2021). As expected, men had significantly greater body mass, height, waist circumference (data not shown) and FFM than women (all *p* < 0.0001), although the body fat percentage did not differ.

Previous investigations have confirmed sex‐based differences in substrate utilization at rest and during exercise (Fernández‐Verdejo et al., 2019; Schneiter et al., 1995), with men typically showing higher absolute fat oxidation rates than women. However, when adjusted for FFM, this difference often diminishes or reverses. In our study, the increased relative fat oxidation observed in women during VL20 (*p* = 0.0257) may reflect inherent muscle characteristics and hormonal influences. Possible mechanisms include elevated oestrogen levels, diminished catecholamine responses and inactivation of α‐adrenergic receptors in adipose tissue (Cano et al., 2022).

This sex‐based divergence is particularly relevant to the long‐term energy balance. Low REE has been implicated in weight regain, because it accounts for ≤70% of the total daily energy expenditure in sedentary individuals (Fernández‐Verdejo et al., 2019). Our findings underscore the importance of considering sex and body composition when designing exercise programmes, particularly for interventions focusing on substrate metabolism and energy expenditure. Further research is neededto confirm the hypothesis that sex and body composition play significant roles in substrate oxidation.

The elevated lactate and heart rate observed following VL40 reflect increased glycolytic flux and sympathetic activation, indicating higher cardiovascular and metabolic activity, though within tolerable limits for overweight/obese individuals. These findings align with previous studies by Larsen et al. (1997, 1999) and (Derave et al., 2007), who reported minimal acute effects of moderate‐ to high‐intensity exercise on glycaemic control in populations with type 2 diabetes mellitus or metabolic syndrome. However, these results contrast with those of a recent meta‐analysis by Engeroff et al. (2023), which found that post‐meal exercise significantly reduced postprandial glycaemic excursions in comparison to pre‐meal exercise [standardized mean difference [SMD] = 0.47 (95% confidence interval 0.23, 0.70)]. Notably, the subgroup analysis of participants with type 2 diabetes mellitus in that review revealed non‐significant effects [SMD = 0.24 (95% confidence interval −0.14, 0.62)], which more closely aligns with our findings.

Several methodological factors may explain these discrepancies, including variations in exercise timing relative to meal intake, differences in protocol duration and intensity, and whether glucose was measured in the blood or interstitial fluid. Exercise performed immediately after a meal might have a more significant glycaemic‐lowering effect than exercise done 30–60 min later. Additionally, the temporary suppression of glycaemic excursions due to increased muscle glucose uptake during exercise may not lead to lasting improvements in postprandial glucose handling.

Our findings also challenge the common belief that skeletal muscle maintains enhanced insulin sensitivity for several hours post‐exercise (Civitarese et al., 2005; Derave et al., 2007; Delgado‐Floody et al., 2020). In this study, RT performed in the morning, after breakfast did not significantly affect the glycaemic or lactate responses to subsequent meals over a 4‐hour period. This indicates that, in overweight or obese individuals, acute RT might not provide immediate metabolic benefits in controlling postprandial hyperglycaemia.

However, caution is warranted when extrapolating acute responses to chronic adaptations. Although the present results do not support RT as a short‐term intervention for postprandial glucose control, substantial evidence supports the long‐term metabolic benefits of resistance exercise. Chronic RT has been shown to upregulate genes involved in lipid oxidation and mitochondrial biogenesis, including *CPT1*, *FAT*/*CD36* and *UCP3*, even in the absence of carbohydrate intake (Civitarese et al., 2005; Heden et al., 2015). These adaptations highlight the potential of RT as a sustainable approach for enhancing metabolic health in high‐risk populations.

Finally, our findings emphasize the need to refine the interpretation of intra‐set VL. Although the VL thresholds of 20% and 40% produced distinct repetition volumes, they did not cause significantlydifferent acute metabolic responses in terms of substrate oxidation. Therefore, VL may serve as a marker for neuromuscular fatigue rather than a determinant of metabolic outcomes. Future studies should explore how manipulating VL in chronic RT programmes influences long‐term cardiometabolic adaptations, particularly in clinical populations with obesity, insulin resistance or sarcopenia (Carrillo‐Arango et al., 2023; Delgado‐Floody et al., 2020).

This study had several limitations that warrant consideration. First, the sample size was modest, and all participants were relatively healthy, inactive adults without overt cardiometabolic diseases. However, the applicability of these findings to individuals with established metabolic disorders, cardiovascular disease or sarcopenic obesity remains uncertain. Second, dietary intake was not standardized prior to testing, and variation in habitual macronutrient composition might have affected  postprandial substrate oxidation. Future studies should control or stratify dietary patterns when assessing metabolic responses to RT. Third, key metabolic and hormonal markers, including insulin, incretins, interleukin‐6, tumour necrosis factor‐α and antioxidant status, were not measured. These factors are essential to postprandial glucose regulation and could clarify the mechanistic pathways underlying the observed responses. Fourth, body composition was assessed using bioelectrical impedance analysis, which, although suitable for population studies, lacks the precision of dual‐energy X‐ray absorptiometry. Employing more robust imaging techniques may improve the accuracy of FFM quantification and its relationship to substrate metabolism (Ramírez‐Vélez et al., 2018). Lastly, participants were not formally familiarized with the exercise protocol prior to testing. Nonetheless, to reducelearning effects and ensure proper technique, all participants received a standardized verbal explanation and visual demonstration of the movement patterns immediately before each session and performed two to three submaximal warm‐up repetitions under supervision before initiating the experimental sets.

Despite these limitations, this study had notable strengths. To our knowledge, this is the first study to examine the acute effects of velocity‐based RT protocols, using distinct intra‐set velocity loss thresholds, on postprandial energy metabolism in individuals with excess body weight who are physically inactive from a Latin American cohort. Additionally, the inclusion of measurements at multiple time points provided a detailed characterization of the temporal dynamics of the postprandial metabolic responses.

## 5 CONCLUSION

In conclusion, both velocity‐based RT protocols, using 20% and 40% velocity loss thresholds, producedpositive acute changes in postprandial metabolism among individuals with excess body weight. The higher threshold (VL40) let to greater increases in fat oxidation and more significant improvements in glycaemic control. These results suggest that adjusting contraction intensity via velocity‐based RT can quickly affect metabolic responses in the postprandial period, which makes up a large part of daily life. Such insights could help to develop exercise interventions designed to lower cardiometabolic risk. Future research should focus on exploring the long‐term effects of velocity‐based RT, specially in populations with insulin resistance or type 2 diabetes, and include hormonal and inflammatory markers to better understand the underlying mechanisms and personalize exercise strategies.

## AUTHOR CONTRIBUTIONS

Hugo Alejandro Carrillo‐Arango: Methodology, software, formal analysis, investigation, project administration. Miguel Alejandro Atencio‐Osorio: Conceptualization, methodology, formal analysis, investigation, data curation, writing, visualization, funding acquisition. Leidy Tatiana Ordoñez‐Mora: Methodology, investigation, data curation. Baisuli Benitez‐Gómez: Methodology, investigation, data curation. Mikel Izquierdo: Conceptualization, methodology, investigation, writing, visualization, supervision. Robinson Ramírez‐Vélez: Conceptualization, methodology, formal analysis, investigation, data curation, writing, visualization, funding acquisition. All authors approved the final version of the manuscript and agree to be accountable for all aspects of the work in ensuring that questions related to the accuracy or integrity of any part of the work are appropriately investigated and resolved. All persons designated as authors qualify for authorship, and all those who qualify for authorship are listed.

## CONFLICT OF INTEREST

None declared.

## Data Availability

Some or all datasets generated and analysed during the present study are not publicly available but are available from the corresponding author upon reasonable request.
